# Experiences of team collaboration among intensive care nurses during COVID-19: a qualitative systematic review and meta-synthesis

**DOI:** 10.3389/fpubh.2026.1852917

**Published:** 2026-07-06

**Authors:** Hui Liao, Yan Gao

**Affiliations:** Department of Respiratory and Critical Care Medicine, West China Hospital, Sichuan University, Chengdu, China

**Keywords:** COVID-19, ICU nurses, meta-synthesis, qualitative research, teamwork

## Abstract

**Objective:**

This study included qualitative studies and employed a meta-synthesis to assess the support, challenges, and coping strategies related to teamwork among Intensive Care Unit (ICU) nurses during the Corona Virus Disease 2019 (COVID-19) pandemic.

**Design:**

Qualitative meta-synthesis.

**Methods:**

A systematic search was conducted across five databases: PubMed, Web of Science, Embase, CINAHL, and PsycINFO. The search period covered from inception to December 11, 2025. The study employed the three-stage thematic synthesis approach by Thomas and Harden, aided by the NVivo 14 software.

**Findings:**

A total of 48 qualitative studies were included, and six main themes with thirteen sub-themes were identified: (1) the support and understanding received from colleagues, the team, and management; (2) interpersonal unfamiliarity and tension; (3) instability in the team structure; (4) managerial inadequacies; (5) team adaptability; (6) empowered teamwork.

**Conclusion:**

Beyond the need for psychological support from colleagues, the team, and management, it was found that strengthening organizational resilience was key to improving teamwork.

**Systematic review registration:**

https://www.crd.york.ac.uk/PROSPERO/view/CRD420261281080, identifier PROSPERO (CRD420261281080).

## 1 Introduction

In March 2020, the World Health Organization (WHO) declared Corona Virus Disease 2019 (COVID-19) a pandemic. In the early phase of the pandemic, an estimated 61.9 million global COVID-19 cases were severe, critical, or fatal (1). COVID-19 rapidly became one of the leading causes of death worldwide (2), and many European countries had not yet returned to their pre-pandemic pattern of declining mortality rates (3). Affected by the COVID-19 pandemic, hospitals and healthcare systems were forced to rapidly create or reorganize care environments, particularly intensive care units (ICUs) (4). The surge of COVID-19 patients was managed through models of interprofessional teamwork and team-based care (5, 6). As core members of this collaborative effort, ICU nurses faced a high-intensity, fast-paced environment with constantly changing colleagues and the demanding needs of critically ill patients with unstable conditions (7). Therefore, gaining an in-depth understanding of their support systems, challenges, and coping strategies within teamwork constituted a critical component for optimizing collaborative models in critical care.

Critical care teams were fluid, capable of expanding from a small core team at the bedside to a larger unit (8). This fluidity expanded the roles and responsibilities of ICU nurses, requiring them not only to care for multiple patients simultaneously and coordinate care but also to supervise and assist temporary colleagues (9). Research by Eckerblad et al. (10) found that ICU nurses lost the security of a stable team and had to adapt to constant change while attempting to establish new ways of collaborating. Similarly, Geltmeyer et al. (6) identified that new care delivery models presented challenges to nursing teams, who reported feeling a loss of control over their clinical practice. Costa et al. (11) demonstrated that both interpersonal factors (individual personality traits and interactions among clinicians) and structural factors (unit-level elements affecting work, organization, and management) could positively and negatively influence team effectiveness. Although the teamwork experiences of ICU nurses during the COVID-19 pandemic received considerable attention, the existing body of evidence was limited by heterogeneous national and cultural contexts, small sample sizes, and inconsistent findings, which compromised its generalizability. Consequently, a systematic review and meta-synthesis were warranted to achieve a deeper, integrated understanding of this specific population.

While previous qualitative meta-syntheses have focused on ICU nurses’ psychological experiences, post-traumatic growth, burnout prevalence, or holistic lived experiences (12–16), the present review is the first to systematically synthesise evidence specifically on teamwork. This includes the support mechanisms, interpersonal tensions, structural instability, managerial inadequacies, adaptive adjustments, and collective agency that emerged from ICU nurses’ collaborative work during the COVID-19 pandemic. This review therefore provides new knowledge at the team level, complementing existing individual-level syntheses. We used the PICo framework (Population, phenomenon of Interest, Context) to provide a structured comparison of the scope of this review with previous related syntheses (see Supplementary Table S1).

This study therefore aimed to conduct a comprehensive review and meta-synthesis of qualitative studies exploring the teamwork process of ICU nurses during the COVID-19 pandemic. It sought to elucidate the support they received from interpersonal, team, and managerial sources, the challenges they faced, and the coping strategies they developed to adapt to team dynamics. Ultimately, the goal was to construct a more explanatory and systematic theoretical model to inform future interventions aimed at enhancing team collaboration.

## 2 Methods

This study adhered to the Preferred Reporting Items for Systematic Reviews and Meta-Analyses (PRISMA) guidelines (17). It also followed the Enhancing Transparency of Qualitative Research Synthesis (ENTREQ) statement (18) (see Supplementary Table S2). The protocol was prospectively registered in the International Prospective Register of Systematic Reviews (PROSPERO) in January 2026 (registration number: CRD 420261281080).

### 2.1 Search process

We searched five databases independently: PubMed, Web of Science, Embase, CINAHL, and PsycINFO. The search period spanned from the inception of each database to December 11, 2025, and Boolean logic operators were used to combine terms during the search process. The MeSH terms used in the search included: “Nurses,” “Nursing,” “Intensive Care Units”, “Critical Care Nursing”, “COVID-19,” “SARS-CoV-2,” and “Qualitative Research.” The free-text keywords mainly included: “Nurse*,” “ICU,” “COVID 19”, “SARS Coronavirus 2 Infection”, and “qualitative stud*.” The asterisk “*” is a truncation symbol commonly used in database searching. It allows for the retrieval of all possible variant endings of a root word. The detailed search strategy is provided in Supplementary Table S3.

### 2.2 Eligibility criteria

#### 2.2.1 Inclusion criteria

*Population (P)*: ICU nurses who were involved in teamwork during the COVID-19 pandemic; in mixed-sample studies (e.g., involving redeployed or newly transferred nurses), data specific to ICU nurses could be extracted separately.

*Interest of phenomenon (I)*: The support received, challenges faced, and coping strategies adopted by nurses in the process of teamwork.

*Context (Co)*: Intensive care units in either public or private hospitals.

*Study design (S)*: Qualitative studies, or the qualitative component of mixed-methods studies, from which textual data could be extracted. Eligible methodologies included phenomenology, grounded theory, ethnography, and descriptive qualitative research, among others.

#### 2.2.2 Exclusion criteria

Studies were excluded if they involved: management personnel or nurses newly transferred to the ICU during the pandemic; were published in languages other than English; were quantitative studies, case reports, conference abstracts, or mixed-methods studies from which qualitative data could not be extracted; were studies for which the full text was unavailable; or were systematic reviews or meta-syntheses.

### 2.3 Screening and data extraction

All retrieved records were imported into EndNote 21 for the removal of duplicates. Two researchers independently screened the titles and abstracts. Subsequently, full texts were sought and obtained for records meeting the inclusion criteria. Finally, the two researchers independently read the full texts to assess eligibility for inclusion. Throughout the screening process, any discrepancies were resolved through discussion with a third researcher.

Data extraction included the following items: first author, year of publication, country, research method or theoretical framework, study participants, phenomenon of interest, context, and key findings. During the extraction of “key findings,” we extracted both the direct verbatim quotations from participants and the themes or subthemes as described by the study authors in the Results section of each included study. The entire data extraction process was conducted independently by two researchers. Any disagreements were resolved through consultation with a third researcher.

### 2.4 Quality assessment

Two researchers independently appraised the methodological quality using the Joanna Briggs Institute (JBI) critical appraisal tool for qualitative research (19). This tool comprises ten items, each of which was rated as “Yes,” “No,” or “Unclear,” corresponding to scores of 2, 0, and 1 point, respectively. The total score was then converted into a percentage. Any discrepancies in appraisal were resolved through discussion with a third researcher.

### 2.5 Decision rules for subtheme allocation from a nurse-centred perspective

This review consistently adopts a nurse-centred perspective. The allocation of subthemes was guided by how nurses themselves experienced, interpreted, and responded to the challenges of the pandemic. The following decision rules were applied to allocate subthemes to the three overarching categories (support, challenges, and coping strategies): (1) Support: factors, resources, or actions that nurses perceived as helpful or enabling for effective teamwork; (2) Challenges: factors, obstacles, or conditions that nurses perceived as hindering or distressing; (3) Coping strategies: actions, responses, or adjustments that nurses actively initiated or engaged in to manage or overcome challenges.

### 2.6 Data analysis and synthesis

Several methods exist for conducting meta-synthesis, a systematic review approach that involves categorizing and re-categorizing the combined findings of two or more primary studies. This review employed the three-stage thematic synthesis approach developed by Thomas and Harden (20). In the first stage, we performed line-by-line coding of the reported meanings and content from the primary studies. In the second stage, based on the codes generated in stage one, we synthesized those with similar meanings to develop descriptive themes. In the third stage, building on the interpretation of the descriptive themes, we engaged in iterative discussions to pose analytical questions for each descriptive theme. Additionally, we compared descriptive themes across studies to identify emerging higher-order concepts, insights, or hypotheses. The process was iterative and continued until the newly developed analytical themes provided a sufficiently clear, explanatory framework for the initial descriptive themes. The analysis was conducted with the aid of NVivo 14 software. Three researchers performed all stages independently. Any disagreements that arose were resolved through ongoing discussion and comparative analysis until a consensus was reached.

### 2.7 Reflexivity

As the perspectives, academic backgrounds, work experiences, and assumptions of the research team could have influenced various stages of the synthesis process, reflexivity played a crucial role (21). LHH and GY are frontline registered nurses working in the respiratory intensive care unit (ICU) of a tertiary hospital in China. Both have direct clinical experience in caring for patients with severe COVID-19 during the pandemic. This insider perspective provided a deep understanding of the clinical context. Our prior assumption about teamwork in the ICU was that “effective collaboration is essential for safe patient care”. Therefore, to reduce bias, we maintained reflexive journals throughout the analysis process to document our initial assumptions and how they evolved. All researchers participated in every stage of the review following training in meta-synthesis methodology, and several members possessed prior qualitative research experience. To mitigate researcher bias and ensure methodological rigor, any uncertainties encountered during the appraisal process were discussed and resolved in weekly team meetings. Furthermore, through a critical realist lens, the team engaged in critical discussions regarding each other’s coding decisions and the construction of themes.

## 3 Results

### 3.1 Literature search

The initial search yielded 3,841 records. After removing duplicates and records excluded for other reasons, 1,865 records remained. Screening of titles and abstracts led to the exclusion of 1,755 records, leaving 110 records for full-text retrieval. Full texts of 6 records could not be obtained despite attempts at retrieval through interlibrary loan, contacting the corresponding authors, and supplementary searches on other academic platforms. Following a full-text review of 104 studies, a final set of 48 studies was included (6, 10, 11, 22–65) (see Supplementary Figure S1).

### 3.2 Study characteristics

The basic characteristics of the 48 included studies are presented in Supplementary Table S4. These studies were published between 2021 and 2025, involving a total of 923 ICU nurses. The studies were conducted in the following countries: the United States (*n* = 7), Turkey (*n* = 7), Iran (*n* = 6), the United Kingdom (*n* = 5), Sweden (*n* = 4), Spain (*n* = 2), South Africa (*n* = 2), Canada (*n* = 2), China (*n* = 2), Greece (*n* = 1), the United Kingdom and Ireland (*n* = 1), Indonesia (*n* = 1), Saudi Arabia (*n* = 1), Thailand (*n* = 1), Australia (*n* = 1), Belgium (*n* = 1), the United Arab Emirates (*n* = 1), Israel (*n* = 1), Norway (*n* = 1), Kuwait (*n* = 1), and Italy (*n* = 1). The predominant methodology was descriptive qualitative research (*n* = 20). The primary data collection method was semi-structured interviews (*n* = 31), and the primary data analysis method was thematic analysis (*n* = 16).

### 3.3 Quality assessment

Two researchers appraised the methodological quality of the 48 included studies using the JBI critical appraisal tool. Any discrepancies that arose during this process were resolved through discussion with a third researcher until consensus was reached. All included studies achieved a quality score of at least 75%, and no study was excluded based on quality. No study was rated as “No” or “Unclear” on Domains 1 to 5 (methodological congruence) or Domains 8 to 10 (participant voice, ethical approval, and linkage of conclusions to data). 35 studies (72.9%) were rated as “No” on Domain 6, indicating that they did not explicitly locate the researcher culturally or theoretically; and 35 studies (72.9%) were rated as “Unclear” on Domain 7, indicating that reflexivity was not clearly reported (see Supplementary Table S5).

### 3.4 Meta-synthesis of qualitative studies

A total of 172 illustrative quotes were extracted from the 48 studies included in this meta-synthesis. These quotes were synthesized into six main themes related to support received, challenges faced, and coping strategies employed: (1) the support and understanding received from colleagues, the team, and management; (2) interpersonal unfamiliarity and tension; (3) instability in the team structure; (4) managerial inadequacies; (5) adaptive adjustments to work patterns; and (6) enhanced both individual and team agency. The themes were further organized into 13 meaningful sub-themes (see Supplementary Figure S2).

#### 3.4.1 Theme 1: the support and understanding received from colleagues, the team, and management

##### 3.4.1.1 Subthemes 1: positive internal team relationships

The shared experience of responding to the COVID-19 pandemic fostered greater mutual understanding and support among colleagues. This dynamic was observed most frequently among nurses (38, 51, 53, 56, 60–64), followed by interactions between nurses and new colleagues (10, 41, 46), and between nurses and physicians (45). ICU nurses actively shared experiences and knowledge, which promoted teamwork. This appeared to become an implicit norm, making mutual support feel essential. For newly joined colleagues, ICU nurses expressed gratitude for their arrival, which alleviated work pressure and boosted team morale. Furthermore, by sharing critical work insights, nurses earned the trust and recognition of physicians.

##### 3.4.1.2 Subthemes 2: teamwork communication synergy

During the COVID-19 pandemic, ICU nurses took pride in themselves and their teams as they worked alongside colleagues to fulfill challenging duties (6, 10, 26, 28, 29, 37, 48, 52). There was a reported desire to enhance cohesion by introducing diverse team-building activities (43). The adoption of adaptive care models was also cited as a way to improve both care effectiveness and intra-team coordination (46). Effective verbal and non-verbal communication within the team not only facilitated collaboration but also alleviated feelings of isolation among ICU nurses, thereby enhancing their sense of autonomous practice (26, 27, 32, 34, 52).

##### 3.4.1.3 Subthemes 3: empowering management

Under intense work pressure, management helped alleviate the workload for ICU nurses by organizing skill-based training (42, 43, 47), establishing specialized functional teams, and sharing up-to-date information (29, 43). Furthermore, by valuing nurses’ needs and engaging in effective communication, management fostered a positive atmosphere that strengthened nurses’ sense of professional efficacy (42).

#### 3.4.2 Theme 2: interpersonal unfamiliarity and tension

##### 3.4.2.1 Subthemes 1: role overload and ambiguity among ICU nurses

The primary cause of role overload reported by ICU nurses was the added responsibility of training new colleagues (10, 22, 27, 42, 47, 50, 54, 65, 66) and helping them integrate and adapt quickly (46). Delegating tasks to new colleagues was described as challenging (11, 41, 45, 59), accompanied by concerns that it might compromise care quality standards (37). Beyond their primary role as care providers, nurses also had to assume supervisory responsibilities. They frequently experienced difficulty and ambiguity in their shifting roles due to having to accept delegations that were unsuitable or outside their expertise (6).

##### 3.4.2.2 Subthemes 2: a loss of interprofessional trust and autonomy

While tension also arose among nurses due to excessive stress (23), a more significant concern was the challenge posed by relationships with new colleagues and physicians. Difficult and unfamiliar interactions with new colleagues implicitly increased the workload (11, 25, 29, 33, 36, 39, 55). Distrust from physicians, often manifesting in orders that increased nurses’ exposure risk and in unreasonable care guidelines, frequently served as a trigger for conflict (11, 24, 50, 57). This led nurses to feel disrespected and frustrated (23, 24, 50). Encounters with irresponsible physicians were reported to cause a sharp increase in stress (22).

#### 3.4.3 Theme 3: instability in the team structure

##### 3.4.3.1 Subthemes 1: systemic disorganization within the team

In the face of the sudden COVID-19 pandemic, teams lacked organizational coordination, resulting in chaotic situations during actual practice (10, 25, 49). Colleagues also lacked the time and opportunity to communicate and collaborate, often leaving problems unresolved promptly (35). This phenomenon was reported to be more common in interprofessional teams (60).

##### 3.4.3.2 Subthemes 2: a breakdown in team structure

The original ICU teams were redeployed, causing nurses to lose their familiar and secure work environment (10, 58). To adapt to the new team composition, they had to collaborate with new colleagues, regardless of whether those colleagues had received adequate training (25). This situation made ICU nurses long for their pre-pandemic teams (29, 37).

#### 3.4.4 Theme 4: managerial inadequacies

##### 3.4.4.1 Subthemes 1: managerial dysfunction

Contradictory protocols and constantly changing tasks issued by management generated dissatisfaction and suspicion among ICU nurses, leading to a decline in trust toward management (10, 30, 31, 47, 61, 66, 67). Furthermore, management assigned tasks beyond nurses’ primary care duties, placing a particularly heavy burden on competent nurses (31, 46, 60).

##### 3.4.4.2 Subthemes 2: resource based oppression

ICU nurses called for an increase in nursing staff, highlighting managerial inadequacies in workforce regulation (11, 30). Furthermore, unreasonable shift schedules left nurses with insufficient time to debrief on work or train new colleagues, creating a vicious cycle (30, 61). A lack of support in the form of bonuses and equipment was also reported (30, 32).

##### 3.4.4.3 Subthemes 3: lack of support and recognition

ICU nurses on the front lines of the COVID-19 pandemic reported receiving insufficient attention and recognition from management, and in some instances, even experienced differential treatment (31, 33, 40, 44, 66). This left nurses, who already expected supportive actions from management, feeling abandoned. Management appeared to be concerned primarily with the work capacity of ICU nurses while neglecting their need for emotional support (23).

#### 3.4.5 Theme 5: team adaptability

##### 3.4.5.1 Subthemes 1: reasonable arrangement of work content and schedule

Through ongoing teamwork, ICU nurses gradually understood the capabilities of new colleagues and attempted to assign appropriate responsibilities and tasks to improve the quality and safety of care (10). Flexible and fair scheduling emerged as a key factor in retaining ICU nurses within the team. They placed particular importance on elements such as part-time positions, optimal shift lengths, shift assignments, and the ability to take approved leave (43).

##### 3.4.5.2 Subthemes 2: collaborative method innovation

ICU nurses progressively mastered the care and management of critically ill COVID-19 patients and attempted to establish new structures and reflect on novel ways of collaborating (10).

#### 3.4.6 Theme 6: empowered teamwork

##### 3.4.6.1 Subthemes 1: knowledge based autonomy

ICU nurses recognized that possessing scientific knowledge was a fundamental requirement for gaining autonomy and respect within the critical care team (32). Making choices based on scientific evidence, especially when navigating difficulties with other professionals, allowed them to secure greater influence during teamwork.

##### 3.4.6.2 Subthemes 2: team focused training

In addition to individual nurses recognizing the importance of their competence within the team, the teams themselves also emphasized training for ICU nurses. This included strengthening skills training for new nurses and conducting regular training on medical equipment use and standardized care procedures (11, 27). This laid the foundation for building reliable collaborative capabilities and enhanced team stability.

## 4 Discussion

This study represents the first systematic review employing meta-synthesis to examine the support, challenges, and coping strategies related to teamwork among ICU nurses during the COVID-19 pandemic. A total of 48 qualitative studies were included. The main findings indicated that the primary support for ICU nurses stemmed from understanding and assistance among colleagues, effective intra-team communication and collaboration, and empowering management. Key challenges included role overload and ambiguity, breakdowns in team structure and organizational chaos, as well as a lack of managerial recognition and unreasonable task allocation. The identified coping strategies involved team adaptability, empowered teamwork, and regular team-organized training.

### 4.1 Support for ICU nurses in teamwork during the COVID-19 pandemic

Our synthesis revealed that ICU nurses received support from three interconnected sources: colleagues, the team itself, and management. These sources functioned in a coordinated manner to maintain both individual and team stability and resilience. As the primary frontline in treating critically ill COVID-19 patients, nurses worked not only in physically isolated environments but also bore the psychological burden of social separation (68). The understanding and practical help provided by colleagues significantly met nurses’ emotional needs and strengthened their sense of team belonging (69). They took pride in being part of a team, perceiving its wholeness and their own value within it (37). Consistent with this, Sun et al. (70) found that nurses experienced a heightened sense of collective strength and team cohesion when confronting the pandemic.

Furthermore, management attempted to implement new strategies to mitigate burnout among ICU nurses. Examples included forming specialized teams for patient repositioning or for Spanish-language communication with patients and families (11). Psychological first aid (PFA), through proactive communication, interpersonal engagement, and resource provision, was also utilized to help create a psychologically safe environment for ICU nurses (71). Therefore, valuing and providing multidimensional support for nurses helped boost their morale and efficiency, which was ultimately translated into a renewed passion for patient care, thereby establishing a positive reinforcing cycle.

### 4.2 Challenges faced by ICU nurses in teamwork during the COVID-19 pandemic

Correspondingly, challenges, which mirrored the dimensions of support, created a cascading impact across three levels: individual role perception, team collaborative processes, and managerial attitudes and organizational capacity. This impact collectively threatened the teamwork capability of ICU nurses. Nurses endured the dual pressure of role overload and ambiguity. The constant influx of critically ill COVID-19 patients, repeatedly changing care models and protocols, the need to collaborate with unfamiliar new colleagues and teams, the responsibility of training these new colleagues, and being assigned unsuitable tasks—all of these factors increased the role burden and distress for ICU nurses (72, 73). Consequently, there was a clear need to define nursing responsibilities and standardize care processes. Cadge et al. (29) found that role clarification could play a vital role in teamwork.

Grounded in transition theory, ICU nurses initially faced chaos during the early phase of the pandemic (74). This chaotic situation manifested in deficiencies in team organization and in communication barriers among colleagues, particularly in interprofessional collaboration and communication. However, over time, ICU nurses learned to navigate this chaos and gradually gained experience in teamwork.

Among these numerous challenges, managerial indifference and disorganized capacity emerged as a leading cause of post-pandemic attrition among ICU nurses (61). This situation was demonstrably detrimental to teamwork in a context of severe nursing shortages. Tracing these challenges to their root cause suggested that organizational chaos and failures in team management likely constituted the foundational issues affecting collaboration. Therefore, it is recommended that future research, when developing intervention programs, should reduce its excessive focus on the “stress tolerance” or “resilience” of ICU nurses as the primary solution.

### 4.3 Coping strategies employed by ICU nurses in teamwork during the COVID-19 pandemic

At the individual level, against the backdrop of role pressure and ambiguity, ICU nurses gradually recognized the importance of professional autonomy. However, the legitimate exercise of this autonomy required a complex knowledge base (75). Acquiring such knowledge not only boosted nurses’ confidence in their work but also helped them earn the respect of other colleagues, a factor that was particularly significant within interprofessional teams. Drawing on Conservation of Resources theory, Xue et al. (76) found that when team insecurity was salient, it motivated employees to engage in self-improvement to protect themselves, thereby increasing their investment in team tasks.

At the team level, teams characterized by high task interdependence relied on each other’s expertise, information, and resources to collectively confront difficulties and enhance collaboration (77). By organizing regular training sessions, teams empowered both individual professional skills and team practice. Additionally, they established structural solutions by streamlining workflows, management practices, protocols, and resource allocation.

From a managerial perspective, conventional scheduling models proved inadequate in the face of the high-pressure work patterns and human resource shortages of the COVID-19 pandemic. Adaptive adjustments to shift schedules and granting nurses the right to take approved leave were identified as crucial reasons for ICU nurses choosing to remain in their roles (43). Following an initial break-in period, assigning new colleagues to suitable tasks enabled them to fulfill vital functions within the team.

Therefore, through proactive and flexible coping strategies implemented across individual, team, and managerial levels, team resilience was enhanced, enabling the effective accomplishment of team-based practice.

### 4.4 Implications for nursing care quality

From the perspective of nursing practice, this study provided the following practical implications. First, given that effective communication (Theme 2), collaboration (Theme 2; Theme 3), and empowering management (Theme 1; Theme 6) were identified as key factors facilitating teamwork, hospitals were advised to institutionalize these elements. This could be achieved through the implementation of daily huddles (Theme 2), structured handoff tools (e.g., SBAR) (Theme 2), and shared decision-making models (Theme 1; Theme 6). Managers were recommended to receive training (Theme 4) that enabled them to empower nurses by delegating authority (Theme 1; Theme 6) and fostering psychological safety (Theme 1), which could enhance team resilience during crises. Second, to address role overload, ambiguity, and structural breakdowns (Theme 3), healthcare institutions were encouraged to further clarify role delineation (Theme 2; Theme 3) and develop contingency plans (Theme 4; Theme 5). Standardized protocols for redeployment (Theme 3; Theme 4), regular team debriefings (Theme 1; Theme 5), and adaptive staffing models (Theme 3; Theme 5) were suggested to reduce chaos. Simulation drills (Theme 2; Theme 5) were proposed to prepare nurses for high-stress scenarios, thereby minimizing confusion and improving role clarity during real emergencies. Finally, as nurses relied on adaptive adjustments to work patterns (Theme 5) and team-organized training (Theme 1; Theme 5), hospitals were recommended to integrate these coping strategies into routine professional development. Regular scenario-based team training (Theme 2; Theme 5), peer support systems(Theme 1), and flexible shift scheduling (Theme 3; Theme 5) were identified as measures that could help nurses build both technical (Theme 5) and emotional coping skills (Theme 1). The implementation of these measures was considered to strengthen teamwork among ICU nurses in future health emergencies.

### 4.5 Transferability

This review included studies from 12 countries across four continents, representing diverse health system structures, economic conditions, and cultural contexts. While this diversity enriches our findings, it also raises important questions about transferability. Themes such as the support and understanding received from colleagues, the team, and management; managerial inadequacies; and empowered teamwork may require consideration of contextual specificity. Regarding the support and understanding received from colleagues, the team, and management, in high-income countries this often took the form of formal psychological support programmes, whereas in low- and middle-income countries (LMICs) it was more frequently expressed as peer-based emotional support. Regarding managerial inadequacies, in high-income settings these often manifested as lack of transparent communication and frequent protocol changes, whereas in LMICs they more often appeared as lack of basic safety infrastructure and absence of visible managerial presence. Regarding empowered teamwork, while the trend toward nurses gaining greater decision-making autonomy appears universal, the degree of empowerment may be influenced by hierarchical medical cultures. In contrast, themes such as interpersonal unfamiliarity and tension, instability in the team structure, and team adaptability appear to be more universal across ICU settings.

### 4.6 Limitations

This study had several limitations. First, it focused exclusively on the support, challenges, and coping strategies related to teamwork among ICU nurses during the COVID-19 pandemic. The perspectives of nursing management and newly recruited ICU nurses were not considered, which may have affected the comprehensiveness of the findings. Second, only studies published in English were included, potentially introducing a language or publication bias. Third, we did not perform sensitivity analyses to exclude studies with lower methodological quality. This is because, on the one hand, the JBI Critical Appraisal Checklist for Qualitative Research is not designed for numeric summation. On the other hand, excluding studies with lower total scores or lower ratings on individual domains would have substantially reduced the diversity of geographical and cultural contexts covered in this review. Fourth, qualitative synthesis inherently involves interpretive subjectivity. Although we enhanced rigour through independent coding, team consensus, iterative discussion, and reflexive journaling, the processes of coding and theme development inevitably reflect the research team’s professional backgrounds, clinical experiences, and theoretical lenses. Therefore, this interpretive subjectivity should be considered when assessing our findings. Finally, due to variations in healthcare systems, economic conditions, political structures, and cultural backgrounds across the included studies, a degree of heterogeneity was likely present among them. Therefore, the findings of this synthesis should be interpreted with caution considering these contextual factors.

### 4.7 Future research directions

Future research directions could be extended to propose specific intervention designs that could be evaluated quantitatively, informed by the theoretical model proposed in this review. For example, team-based crisis training could be developed and tested based on the themes of team adaptability and empowered teamwork, evaluating the effects of such interventions on team functioning, role clarity, and nurse well-being during surge events. As another example, managerial support interventions could be designed based on the themes of managerial inadequacies, assessing the impact of manager training on team resilience and perceived organisational support.

## 5 Conclusion

This meta-synthesis provided key insights for enhancing the teamwork capacity of ICU nurses during the COVID-19 pandemic. It offered an in-depth analysis of the support received, challenges faced, and coping strategies adopted by ICU nurses within the context of pandemic teamwork, examined through the individual, team, and managerial levels. Building upon the provision of adequate psychological support for ICU nurses, strengthening the team’s organizational adaptive capacity emerged as a crucial element for improving collaborative performance.

## Data Availability

The original contributions presented in the study are included in the article/Supplementary material, further inquiries can be directed to the corresponding author.
