# Supplementary material for: Experiences of team collaboration among intensive care nurses during COVID-19: a qualitative systematic review and meta-synthesis
Source: Front Public Health. 2026 Jul 6;14:1852917. doi: 10.3389/fpubh.2026.1852917 (PMC13381427; doi:10.3389/fpubh.2026.1852917)
Supplement: Supplementary file 1 [file Data_Sheet_1.DOCX]

**Table S1Comparison of the present review with prior qualitative meta‑syntheses on ICU nurses during the COVID‑19 pandemic (PICo framework).**

| **Review** | **Population (P)** | **Phenomenon of Interest (I)** | **Context (Co)** | **Main outcomes/focus** |
| --- | --- | --- | --- | --- |
| Han et al. (2022) | ICU nurses caring for severe COVID‑19 patients | Psychological experiences (mood, cognitive‑behavioural responses, social factors) | ICUs during COVID‑19 pandemic | **Three themes:** physical reactions and psychological changes; need for support from multiple sources; increased adaptation and resilience |
| Liu et al. (2024) | Emergency and critical care (ECC) nurses | Post‑traumatic growth (PTG) after the pandemic | ECC units after COVID‑19 pandemic | **Three themes:** stress period, adjustment period and growth period. Eight sub-themes: negative emotion, psychological gap, self adjusting, social support, improvement of personal ability, increased sense of professional belonging, spiritual awakening and extended thinking, look ahead. |
| Nasrabadi et al. (2022) | ICU nurses working with COVID‑19 patients | Holistic experiences (not limited to teamwork) | ICUs during COVID‑19 pandemic | **Six themes:** distance from holistic nursing; psychosocial experiences; efforts for self‑protection; organisational inefficiency; job burnout; emerging new experiences in the workplace |
| Papazian et al. (2023) | ICU physicians and nurses | Burnout prevalence (quantitative meta‑analysis with qualitative elements) | Adult ICUs | Prevalence of high‑level burnout (41% in physicians, **44%** in nurses); higher emotional exhaustion in nurses |
| Rourke et al. (2024) | Critical care nurses (CCNs) | Lived experiences (broad, including teamwork as one of several themes) | ICUs during COVID‑19 pandemic | **Eight themes:** teamwork, patient‑centred care, death, family support, psychological impact, ICU challenges, PPE challenges, impact on home life |
| **Present review** | ICU nurses (mixed‑sample studies included only if ICU nurse data extractable) | **Teamwork‑specific experiences**: support received, challenges faced, and coping strategies in teamwork | ICUs (public or private hospitals) during COVID‑19 pandemic | **Six themes:** (1) support from colleagues/team/management; (2) interpersonal unfamiliarity and tension; (3) instability in team structure; (4) managerial inadequacies; (5) adaptive adjustments to work patterns; (6) enhanced individual and team agency |

**Table S2 PRISMA 2020 checklist.**

| **Section and Topic** | **Item #** | **Checklist item** | **Location where item is reported** |
| --- | --- | --- | --- |
| **TITLE** | | |  |
| Title | 1 | Identify the report as a systematic review. | 1 |
| **ABSTRACT** | | |  |
| Abstract | 2 | See the PRISMA 2020 for Abstracts checklist. | 1 |
| **INTRODUCTION** | | |  |
| Rationale | 3 | Describe the rationale for the review in the context of existing knowledge. | 2 |
| Objectives | 4 | Provide an explicit statement of the objective(s) or question(s) the review addresses. | 2 |
| **METHODS** | | |  |
| Eligibility criteria | 5 | Specify the inclusion and exclusion criteria for the review and how studies were grouped for the syntheses. | 3 |
| Information sources | 6 | Specify all databases, registers, websites, organisations, reference lists and other sources searched or consulted to identify studies. Specify the date when each source was last searched or consulted. | 3 |
| Search strategy | 7 | Present the full search strategies for all databases, registers and websites, including any filters and limits used. | 3 |
| Selection process | 8 | Specify the methods used to decide whether a study met the inclusion criteria of the review, including how many reviewers screened each record and each report retrieved, whether they worked independently, and if applicable, details of automation tools used in the process. | 3 |
| Data collection process | 9 | Specify the methods used to collect data from reports, including how many reviewers collected data from each report, whether they worked independently, any processes for obtaining or confirming data from study investigators, and if applicable, details of automation tools used in the process. | 3 |
| Data items | 10a | List and define all outcomes for which data were sought. Specify whether all results that were compatible with each outcome domain in each study were sought (e.g. for all measures, time points, analyses), and if not, the methods used to decide which results to collect. | 3 |
|  | 10b | List and define all other variables for which data were sought (e.g. participant and intervention characteristics, funding sources). Describe any assumptions made about any missing or unclear information. | 4 |
| Study risk of bias assessment | 11 | Specify the methods used to assess risk of bias in the included studies, including details of the tool(s) used, how many reviewers assessed each study and whether they worked independently, and if applicable, details of automation tools used in the process. | 4 |
| Effect measures | 12 | Specify for each outcome the effect measure(s) (e.g. risk ratio, mean difference) used in the synthesis or presentation of results. | 4 |
| Synthesis methods | 13a | Describe the processes used to decide which studies were eligible for each synthesis (e.g. tabulating the study intervention characteristics and comparing against the planned groups for each synthesis (item #5)). | 4 |
|  | 13b | Describe any methods required to prepare the data for presentation or synthesis, such as handling of missing summary statistics, or data conversions. | 4 |
|  | 13c | Describe any methods used to tabulate or visually display results of individual studies and syntheses. | 4 |
|  | 13d | Describe any methods used to synthesize results and provide a rationale for the choice(s). If meta-analysis was performed, describe the model(s), method(s) to identify the presence and extent of statistical heterogeneity, and software package(s) used. | 4 |
|  | 13e | Describe any methods used to explore possible causes of heterogeneity among study results (e.g. subgroup analysis, meta-regression). | 4 |
|  | 13f | Describe any sensitivity analyses conducted to assess robustness of the synthesized results. | 4 |
| Reporting bias assessment | 14 | Describe any methods used to assess risk of bias due to missing results in a synthesis (arising from reporting biases). | 4 |
| Certainty assessment | 15 | Describe any methods used to assess certainty (or confidence) in the body of evidence for an outcome. | 4 |
| **RESULTS** | | |  |
| Study selection | 16a | Describe the results of the search and selection process, from the number of records identified in the search to the number of studies included in the review, ideally using a flow diagram. | 4 |
|  | 16b | Cite studies that might appear to meet the inclusion criteria, but which were excluded, and explain why they were excluded. | 4 |
| Study characteristics | 17 | Cite each included study and present its characteristics. | 4 |
| Risk of bias in studies | 18 | Present assessments of risk of bias for each included study. | 4 |
| Results of individual studies | 19 | For all outcomes, present, for each study: (a) summary statistics for each group (where appropriate) and (b) an effect estimate and its precision (e.g. confidence/credible interval), ideally using structured tables or plots. | 5 |
| Results of syntheses | 20a | For each synthesis, briefly summarise the characteristics and risk of bias among contributing studies. | 5 |
|  | 20b | Present results of all statistical syntheses conducted. If meta-analysis was done, present for each the summary estimate and its precision (e.g. confidence/credible interval) and measures of statistical heterogeneity. If comparing groups, describe the direction of the effect. | 5 |
|  | 20c | Present results of all investigations of possible causes of heterogeneity among study results. | 5 |
|  | 20d | Present results of all sensitivity analyses conducted to assess the robustness of the synthesized results. | 5 |
| Reporting biases | 21 | Present assessments of risk of bias due to missing results (arising from reporting biases) for each synthesis assessed. | 6 |
| Certainty of evidence | 22 | Present assessments of certainty (or confidence) in the body of evidence for each outcome assessed. | 6 |
| **DISCUSSION** | | |  |
| Discussion | 23a | Provide a general interpretation of the results in the context of other evidence. | 7 |
|  | 23b | Discuss any limitations of the evidence included in the review. | 7 |
|  | 23c | Discuss any limitations of the review processes used. | 8 |
|  | 23d | Discuss implications of the results for practice, policy, and future research. | 9 |
| **OTHER INFORMATION** | | |  |
| Registration and protocol | 24a | Provide registration information for the review, including register name and registration number, or state that the review was not registered. | 3 |
|  | 24b | Indicate where the review protocol can be accessed, or state that a protocol was not prepared. | 3 |
|  | 24c | Describe and explain any amendments to information provided at registration or in the protocol. | 3 |
| Support | 25 | Describe sources of financial or non-financial support for the review, and the role of the funders or sponsors in the review. | 10 |
| Competing interests | 26 | Declare any competing interests of review authors. | 10 |
| Availability of data, code and other materials | 27 | Report which of the following are publicly available and where they can be found: template data collection forms; data extracted from included studies; data used for all analyses; analytic code; any other materials used in the review. | 10 |

*From:*  Page MJ, McKenzie JE, Bossuyt PM, Boutron I, Hoffmann TC, Mulrow CD, et al. The PRISMA 2020 statement: an updated guideline for reporting systematic reviews. BMJ 2021;372:n71. doi: 10.1136/bmj.n71

For more information, visit: <http://www.prisma-statement.org/>

**Table S3: Search strategy**

**Pubmed**

| Search number | Search Details | Results |
| --- | --- | --- |
| 8:5AND6AND7 | (((Qualitative Research[MeSH Terms]) OR (Qualitative Research[Title/Abstract] OR qualitative stud*[Title/Abstract] OR Grounded theory[Title/Abstract] OR interview[Title/Abstract] OR phenomenology[Title/Abstract] OR Content analysis[Title/Abstract] OR Case analysis[Title/Abstract] OR action research[Title/Abstract] OR ethnography[Title/Abstract] OR experiences[Title/Abstract] OR perceptions[Title/Abstract] OR attitudes[Title/Abstract] OR views[Title/Abstract] OR feelings[Title/Abstract] OR perspectives[Title/Abstract] OR opinions[Title/Abstract])) AND (((COVID-19[MeSH Terms]) OR (SARS-CoV-2[MeSH Terms])) OR (COVID 19[Title/Abstract] OR SARS Coronavirus 2 Infection[Title/Abstract] OR 2019 nCoV Infection*[Title/Abstract] OR SARS CoV 2 Infection[Title/Abstract] OR 2019 Novel Coronavirus Disease[Title/Abstract] OR 2019 Novel Coronavirus Infection[Title/Abstract] OR 2019 nCoV Disease*[Title/Abstract] OR Coronavirus Disease 2019[Title/Abstract] OR Severe Acute Respiratory Syndrome Coronavirus 2 Infection[Title/Abstract] OR Coronavirus Disease 19[Title/Abstract] OR Wuhan Coronavirus[Title/Abstract] OR Wuhan Seafood Market Pneumonia Virus[Title/Abstract] OR Coronavirus Disease 2019 Virus[Title/Abstract] OR Severe Acute Respiratory Syndrome Coronavirus 2[Title/Abstract] OR SARS Coronavirus 2[Title/Abstract] OR 2019-nCoV[Title/Abstract] OR SARS CoV 2 Virus[Title/Abstract] OR 2019 Novel Coronavirus*[Title/Abstract]))) AND (((((Nurses[MeSH Terms]) OR (Nursing[MeSH Terms])) OR (Nurse*[Title/Abstract] OR Nursing*[Title/Abstract] OR Nursing Personnel[Title/Abstract] OR Registered Nurse*[Title/Abstract])) AND ((Intensive Care Units[MeSH Terms]) OR (Intensive Care Unit*[Title/Abstract] OR ICU[Title/Abstract] OR Critical Care[Title/Abstract] OR Intensive Care[Title/Abstract] OR Critical Care Nursing[Title/Abstract] OR Critical Care Unit*[Title/Abstract] OR CCU[Title/Abstract] OR close attention unit[Title/Abstract] OR intensive care department[Title/Abstract] OR intensive therapy unit[Title/Abstract] OR intensive treatment unit[Title/Abstract] OR respiratory care unit*[Title/Abstract] OR special care unit[Title/Abstract]))) OR ((Critical Care Nursing[MeSH Terms]) OR (Intensive Care Nursing[Title/Abstract] OR critical care nursing[Title/Abstract] OR ICU nursing[Title/Abstract] OR intensive care nursing[Title/Abstract]))) | 524 |
| 7 | (Qualitative Research[MeSH Terms]) OR (Qualitative Research[Title/Abstract] OR qualitative stud*[Title/Abstract] OR Grounded theory[Title/Abstract] OR interview[Title/Abstract] OR phenomenology[Title/Abstract] OR Content analysis[Title/Abstract] OR Case analysis[Title/Abstract] OR action research[Title/Abstract] OR ethnography[Title/Abstract] OR experiences[Title/Abstract] OR perceptions[Title/Abstract] OR attitudes[Title/Abstract] OR views[Title/Abstract] OR feelings[Title/Abstract] OR perspectives[Title/Abstract] OR opinions[Title/Abstract]) | 1,219,307 |
| 6 | ((COVID-19[MeSH Terms]) OR (SARS-CoV-2[MeSH Terms])) OR (COVID 19[Title/Abstract] OR SARS Coronavirus 2 Infection[Title/Abstract] OR 2019 nCoV Infection*[Title/Abstract] OR SARS CoV 2 Infection[Title/Abstract] OR 2019 Novel Coronavirus Disease[Title/Abstract] OR 2019 Novel Coronavirus Infection[Title/Abstract] OR 2019 nCoV Disease*[Title/Abstract] OR Coronavirus Disease 2019[Title/Abstract] OR Severe Acute Respiratory Syndrome Coronavirus 2 Infection[Title/Abstract] OR Coronavirus Disease 19[Title/Abstract] OR Wuhan Coronavirus[Title/Abstract] OR Wuhan Seafood Market Pneumonia Virus[Title/Abstract] OR Coronavirus Disease 2019 Virus[Title/Abstract] OR Severe Acute Respiratory Syndrome Coronavirus 2[Title/Abstract] OR SARS Coronavirus 2[Title/Abstract] OR 2019-nCoV[Title/Abstract] OR SARS CoV 2 Virus[Title/Abstract] OR 2019 Novel Coronavirus*[Title/Abstract]) | 477,082 |
| 5:3OR4 | ((((Nurses[MeSH Terms]) OR (Nursing[MeSH Terms])) OR (Nurse*[Title/Abstract] OR Nursing*[Title/Abstract] OR Nursing Personnel[Title/Abstract] OR Registered Nurse*[Title/Abstract])) AND ((Intensive Care Units[MeSH Terms]) OR (Intensive Care Unit*[Title/Abstract] OR ICU[Title/Abstract] OR Critical Care[Title/Abstract] OR Intensive Care[Title/Abstract] OR Critical Care Nursing[Title/Abstract] OR Critical Care Unit*[Title/Abstract] OR CCU[Title/Abstract] OR close attention unit[Title/Abstract] OR intensive care department[Title/Abstract] OR intensive therapy unit[Title/Abstract] OR intensive treatment unit[Title/Abstract] OR respiratory care unit*[Title/Abstract] OR special care unit[Title/Abstract]))) OR ((Critical Care Nursing[MeSH Terms]) OR (Intensive Care Nursing[Title/Abstract] OR critical care nursing[Title/Abstract] OR ICU nursing[Title/Abstract] OR intensive care nursing[Title/Abstract])) | 37,564 |
| 4 | (Critical Care Nursing[MeSH Terms]) OR (Intensive Care Nursing[Title/Abstract] OR critical care nursing[Title/Abstract] OR ICU nursing[Title/Abstract] OR intensive care nursing[Title/Abstract]) | 5,057 |
| 3:1AND2 | (((Nurses[MeSH Terms]) OR (Nursing[MeSH Terms])) OR (Nurse*[Title/Abstract] OR Nursing*[Title/Abstract] OR Nursing Personnel[Title/Abstract] OR Registered Nurse*[Title/Abstract])) AND ((Intensive Care Units[MeSH Terms]) OR (Intensive Care Unit*[Title/Abstract] OR ICU[Title/Abstract] OR Critical Care[Title/Abstract] OR Intensive Care[Title/Abstract] OR Critical Care Nursing[Title/Abstract] OR Critical Care Unit*[Title/Abstract] OR CCU[Title/Abstract] OR close attention unit[Title/Abstract] OR intensive care department[Title/Abstract] OR intensive therapy unit[Title/Abstract] OR intensive treatment unit[Title/Abstract] OR respiratory care unit*[Title/Abstract] OR special care unit[Title/Abstract])) | 36,806 |
| 2 | (Intensive Care Units[MeSH Terms]) OR (Intensive Care Unit*[Title/Abstract] OR ICU[Title/Abstract] OR Critical Care[Title/Abstract] OR Intensive Care[Title/Abstract] OR Critical Care Nursing[Title/Abstract] OR Critical Care Unit*[Title/Abstract] OR CCU[Title/Abstract] OR close attention unit[Title/Abstract] OR intensive care department[Title/Abstract] OR intensive therapy unit[Title/Abstract] OR intensive treatment unit[Title/Abstract] OR respiratory care unit*[Title/Abstract] OR special care unit[Title/Abstract]) | 332,957 |
| 1 | ((Nurses[MeSH Terms]) OR (Nursing[MeSH Terms])) OR (Nurse*[Title/Abstract] OR Nursing*[Title/Abstract] OR Nursing Personnel[Title/Abstract] OR Registered Nurse*[Title/Abstract]) | 749,609 |

**Embase**

| No. | Query | Results |
| --- | --- | --- |
| #8 | #5 AND #6 AND #7 | 714 |
| #7 | 'qualitative research'/exp OR 'qualitative research':ab,ti OR 'qualitative stud*':ab,ti OR 'grounded theory':ab,ti OR interview:ab,ti OR phenomenology:ab,ti OR 'content analysis':ab,ti OR 'case analysis':ab,ti OR 'action research':ab,ti OR ethnography:ab,ti OR experiences:ab,ti OR perceptions:ab,ti OR attitudes:ab,ti OR views:ab,ti OR feelings:ab,ti OR perspectives:ab,ti OR opinions:ab,ti | 1510792 |
| #6 | 'covid 19'/exp OR 'sars cov 2'/exp OR 'covid 19':ab,ti OR 'sars coronavirus 2 infection':ab,ti OR '2019 ncov infection*':ab,ti OR 'sars cov 2 infection':ab,ti OR '2019 novel coronavirus disease':ab,ti OR '2019 novel coronavirus infection':ab,ti OR '2019 ncov disease*':ab,ti OR 'coronavirus disease 2019':ab,ti OR 'severe acute respiratory syndrome coronavirus 2 infection':ab,ti OR 'coronavirus disease 19':ab,ti OR 'wuhan coronavirus':ab,ti OR 'wuhan seafood market pneumonia virus':ab,ti OR 'coronavirus disease 2019 virus':ab,ti OR 'severe acute respiratory syndrome coronavirus 2':ab,ti OR 'sars coronavirus 2':ab,ti OR '2019 ncov':ab,ti OR 'sars cov 2 virus':ab,ti OR '2019 novel coronavirus*':ab,ti | 602955 |
| #5 | #3 OR #4 | 60151 |
| #4 | 'intensive care nursing'/exp OR 'critical care nursing':ab,ti OR 'icu nursing':ab,ti OR 'intensive care nursing':ab,ti | 5968 |
| #3 | #1 AND #2 | 59205 |
| #2 | 'intensive care unit'/exp OR 'intensive care unit*':ab,ti OR icu:ab,ti OR 'critical care':ab,ti OR 'intensive care':ab,ti OR 'critical care nursing':ab,ti OR 'critical care unit*':ab,ti OR ccu:ab,ti OR 'close attention unit':ab,ti OR 'intensive care department':ab,ti OR 'intensive therapy unit':ab,ti OR 'intensive treatment unit':ab,ti OR 'respiratory care unit*':ab,ti OR 'special care unit':ab,ti | 622758 |
| #1 | 'nurses'/exp OR 'nursing'/exp OR nurse*:ab,ti OR nursing*:ab,ti OR 'nursing personnel':ab,ti OR 'registered nurse*':ab,ti | 966115 |

**Web of Science**

#1: Nurse* OR Nursing* OR Nursing Personnel OR Registered Nurse* (Topic) AND Intensive Care Unit* OR ICU OR Critical Care OR Intensive Care OR Critical Care Nursing OR Critical Care Unit* OR CCU OR close attention unit OR intensive care department OR intensive therapy unit OR intensive treatment unit OR respiratory care unit* OR special care unit (Topic) Results: 39932

#2: Intensive Care Nursing OR critical care nursing OR ICU nursing OR intensive care nursing (Topic) Results: 38094

#3: COVID 19 OR SARS Coronavirus 2 Infection OR 2019 nCoV Infection* OR SARS CoV 2 Infection OR 2019 Novel Coronavirus Disease OR 2019 Novel Coronavirus Infection OR 2019 nCoV Disease* OR Coronavirus Disease 2019 OR Severe Acute Respiratory Syndrome Coronavirus 2 Infection OR Coronavirus Disease 19 OR Wuhan Coronavirus OR Wuhan Seafood Market Pneumonia Virus OR Coronavirus Disease 2019 Virus OR Severe Acute Respiratory Syndrome Coronavirus 2 OR SARS Coronavirus 2 OR 2019-nCoV OR SARS CoV 2 Virus OR 2019 Novel Coronavirus* (Topic)

Results: 620538

#4: Qualitative Research OR qualitative stud* OR Grounded theory OR interview OR phenomenology OR Content analysis OR Case analysis OR action research OR ethnography OR experiences OR perceptions OR attitudes OR views OR feelings OR perspectives OR opinions (Topic)

Results: 620538

#5: #2 OR #3 Results: 39932

#6: #3 AND #4 AND #5 Results: 1774

**CINAHL**

**S1** SU (Nurses OR Nursing) OR AB (Nurse* OR Nursing* OR Nursing Personnel OR Registered Nurse*) Results: 997070

**S2** SU (Intensive Care Units) OR AB (Intensive Care Unit* OR ICU OR Critical Care OR Intensive Care OR Critical Care Nursing OR Critical Care Unit* OR CCU OR close attention unit OR intensive care department OR intensive therapy unit OR intensive treatment unit OR respiratory care unit* OR special care unit) Results: 138791

**S3** S1 AND S2 Results: 35271

**S4** SU (critical care nursing or intensive care nursing) OR AB (Intensive Care Nursing OR critical care nursing OR ICU nursing OR intensive care nursing)

Results: 28686

**S5** S3 OR S4 Results: 49357

**S6** SU (COVID-19 OR SARS-CoV-2) OR AB (COVID 19 OR SARS Coronavirus 2 Infection OR 2019 nCoV Infection* OR SARS CoV 2 Infection OR 2019 Novel Coronavirus Disease OR 2019 Novel Coronavirus Infection OR 2019 nCoV Disease* OR Coronavirus Disease 2019 OR Severe Acute Respiratory Syndrome Coronavirus 2 Infection OR Coronavirus Disease 19 OR Wuhan Coronavirus OR Wuhan Seafood Market Pneumonia Virus OR Coronavirus Disease 2019 Virus OR Severe Acute Respiratory Syndrome Coronavirus 2 OR SARS Coronavirus 2 OR 2019-nCoV OR SARS CoV 2 Virus OR 2019 Novel Coronavirus*) Results: 143534

**S7** SU (Qualitative Research) OR AB (Qualitative Research OR qualitative stud* OR Grounded theory OR interview OR phenomenology OR Content analysis OR Case analysis OR action research OR ethnography OR experiences OR perceptions OR attitudes OR views OR feelings OR perspectives OR opinions) Results: 887929

**S8** S7 AND S6 AND S5 Results:648

**PsycINFO**

| **S#** | **Query (user-entered)** | **Results** |
| --- | --- | --- |
| **S8** | S7 AND S4 AND S5 | 181 |
| **S7** | S6 OR S3 | 6301 |
| **S6** | S1 AND S2 | 6238 |
| **S5** | SU (Qualitative Research) OR AB (Qualitative Research OR qualitative stud* OR Grounded theory OR interview OR phenomenology OR Content analysis OR Case analysis OR action research OR ethnography OR experiences OR perceptions OR attitudes OR views OR feelings OR perspectives OR opinions) | 1910158 |
| **S4** | SU (COVID-19 OR SARS-CoV-2) OR AB (COVID 19 OR SARS Coronavirus 2 Infection OR 2019 nCoV Infection* OR SARS CoV 2 Infection OR 2019 Novel Coronavirus Disease OR 2019 Novel Coronavirus Infection OR 2019 nCoV Disease* OR Coronavirus Disease 2019 OR Severe Acute Respiratory Syndrome Coronavirus 2 Infection OR Coronavirus Disease 19 OR Wuhan Coronavirus OR Wuhan Seafood Market Pneumonia Virus OR Coronavirus Disease 2019 Virus OR Severe Acute Respiratory Syndrome Coronavirus 2 OR SARS Coronavirus 2 OR 2019-nCoV OR SARS CoV 2 Virus OR 2019 Novel Coronavirus*) | 64426 |
| **S3** | SU (critical care nursing or intensive care nursing) OR AB (Intensive Care Nursing OR critical care nursing OR ICU nursing OR intensive care nursing) | 1156 |
| **S2** | SU (Intensive Care Units) OR AB (Intensive Care Unit* OR ICU OR Critical Care OR Intensive Care OR Critical Care Nursing OR Critical Care Unit* OR CCU OR close attention unit OR intensive care department OR intensive therapy unit OR intensive treatment unit OR respiratory care unit* OR special care unit) | 19742 |
| **S1** | SU (Nurses OR Nursing) OR AB (Nurse* OR Nursing* OR Nursing Personnel OR Registered Nurse*) | 146210 |

**Table S4 Characteristics of studies included.**

| First Author (year) | Country | Study design /methods/theory | Participants | Aim | Location | Themes |
| --- | --- | --- | --- | --- | --- | --- |
| Aamodt  (2025) | Norway | Descriptive explorative design; semi-structured interviews; Braun and Clarke's six-phase reflexive thematic analysis | 14 CCNs | Explore CCNs experience of their role and scope of practice when caring for patients with COVID-19 during the pandemic in Norway | 5 ICUs from four hospitals in south-eastern Norway | 3 themes: promoting safe critical care nursing; competence in critical care nursing; the moral responsibility of a critical care nurse. |
| Al Haddad  (2024) | Kuwaiti | Qualitative study; semi-structured interview; Charmaz’s grounded theory | 25 ICU nurses | Elucidate the challenges faced by ICU nurses in Kuwait during the pandemic | Three public hospitals in Kuwait | 2 themes: factors contributing  to the intensification of pressure; the impact upon the nurses.  4 sub-themes: comprised the factors contributing to pressure (more patients, less support; no rest, no recovery; strained relationships; no choice, no voice)  3 sub-themes: comprised the impact on the nurses (compromised care; moral distress; deteriorating mental health) |
| Alzailai  (2023) | Saudi Arabia | Constructivist grounded theory design | 22 ICU nurses | Examine ICU nurses’ experiences during the COVID-19 pandemic in Saudi Arabia to develop insights into the factors that in uenced burnout | An adult ICU in a tertiary hospital in the Makkah province in the Kingdom of Saudi Arabia | Family, work, and the wider world context  are the three groups of contextual factors that in uenced nurses' experience and perception of burnout |
| Askar  (2023) | Turkey | Descriptive qualitative design; semi-structured interviews; thematic analysis | 11 nurses, all working in the newly built COVID-19 intensive care unit for at least 15 days | Explore and describe the caregiving experiences of nurses who were working in a new COVID-19 ICU in Turkey and providing care for patients infected with SARS-CoV-2 | New COVID-19 ICU in a tertiary university hospital in southern Türkiye | 4 themes: uncertainty and challenges in the ‘danger zone’; emotional and psychosocial changes; professional effects of the pandemic; being ready for a future pandemic |
| Aydin  (2022) | Turkey | Descriptive phenomenological research method; semi-structured interviews; theory of self-transcendence | 25 nurses, with an average working time of 3.9 months in the COVID-19 ICU | Examine the self-transcendence of the leading fighters, ICU nurses, during the COVID-19 pandemic | The COVID-19 intensive care service in different provinces of Turkey | 3 themes: findings are discussed under the headings of self-transcendence; well-being; vulnerability |
| Baran  (2024) | Turkey | Empirical phenomenological approach; semi-structured interviews | 50 nurses, working in the COVID-19 ICU for an average of about 8 months | Describe the working experiences, feelings, and thoughts of nurses working in the ICU during the COVID-19 pandemic from their perspective | A hospital in Ankara, Turkey | 6 themes: backbone of the health system nursing; professional achievements; difficulties encountered; support needs and expectations; changes in emotions and private life |
| Besen  (2023) | Turkey | Descriptive qualitative study; Phenomenological type; semi-structured interview | 12 ICU nurses | Reveal the experiences, problems, motivation and support resources of ICU in the first period of the COVID-19 pandemic | ICU of a public hospital in Izmir, Turkey | 6 main themes (sub-themes); emotions (worry/anxiety), difficulties in patient care (aspiration, intubation), measures taken by nurses (internal isolation), effects of the pandemic on intensive care nurses (physical; back pain, psychological; sleep problems, social; exclusion), support and motivation sources of nurses (teammates support), positive contributions of the pandemic process (crisis management) |
| Cadge  (2021) | America | Qualitative research; semi-structured interview | 16 nurses who cared for COVID-19 patients in the ICU consisted of 8 ICU nurses and 8 general ward nurses. (mixed sample) | Explore nurses’ experiences of provid- ing care to patients with COVID-19 in the ICU | A quaternary 1,000-bed hospital during the first COVID-19 pandemic surge in Boston | 4 themes: chal- lenges of working with new co-workers and teams; challenges of maintaining existing working relationships; role of nursing leadership in providing information and maintaining morale; the importance of institutional level acknowledgement of their work |
| Chegini  (2021) | Iran | Descriptive phenomenological research method; semi-structured interviews;7‐step method of Colaizzi | 15 ICU nurses with an average of 17.69 years of work experience | Describe the experiences of CCNs caring for patients infected by COVID-19 | ICUs in Iranian Public Hospitals | 3 themes: a mixture of positive and negative effects on the psychological; social, and professional challenges; the negative effect only on the organizational challenges |
| Christianson  (2022) | America | Descriptive qualitative study; semi-structured interview | 12 ICU nurses | Examine the impact of the COVID-19 pandemic on the duty of care balance among ICU nurses who manage COVID-19 patients | ICU nurses across the United States | 4 themes: perceptions of betrayal or abandonment of nurses; deviations from the normalized standard of care expectations; feeling a moral obligation to care for COVID-19 patients; the expectation of self-sacrifice contributing to the experience of burnout |
| Costa  (2023) | America | Descriptive qualitative study; semi-structured interview | 3 ICU nurses; 3 moderate care nurses (mixed sample) | Explore the perceptions, challenges, and potential improvements regarding team dynamics among healthcare professionals in a newly established COVID-19 ICU | A newly established, 36- to 50-bed adult COVID-19 ICU within a teaching hospital in the United States. | 2 themes: interpersonal factors (individual character traits and interactions among clinicians); structural factors (unit-level factors affecting work ow, organisation, and administration) |
| Costa  (2023) | America | Descriptive qualitative study; semi-structured interview; thematic analysis | 19 ICU nurses with at least 6 months of work experience | To analyze the exercise of professional autonomy of ICU nurses during times of the new coronavirus pandemic | Three large hospitals in Maceyo, Alagoas state, Brazil | 2 themes: requirements for the exercise of professional autonomy in the face of the Covid-19 pandemic; professional autonomy and working conditions in times of pandemic |
| Credland  (2024) | the United Kingdom; Ireland | Qualitative study; semi-structured interview; thematic analysis | 54 ICU nurses | Explore the challenges to mental health and psychological well-being experienced by CCNs during the COVID-19 pandemic | 38 hospitals in the United Kingdom and Ireland | 4 themes: lack of control; psychological trauma; unexpected leadership; public-political betrayal |
| Díaz-Agea  (2022) | Spain | Descriptive qualitative study; semi-structured interview | 6 ICU nurses | Determine the subjective impact of the pandemic due to COVID-19 on communication | Multiple public hospitals in the Murcia region of Spain | 4 themes: communication (communicative expressions, both verbal and non-verbal-, and limitations); emotional aspects (positive, negative); overload (first wave, second wave, and third wave); relationships (health professionals–patients, healthcare professionals, patients family, and family health professionals). |
| Digby  (2023) | Australia | Qualitative exploratory design; Interview method for audio recording and transcription; thematic Analysis | 13 ICU nurses | Explore the experiences and perceptions of ICU staff regarding patient care and communication during the COVID-19 pandemic | A 56-bed ICU in a major metropolitan teaching hospital in Australia. | 4 themes: communication and connection; psychological casualties; caring for our patients; overcoming challenges. |
| Eckerblad  (2025) | Sweden | Qualitative research; Semi-structured interview; Braun & Clarke’s six-step inductive thematic analysis | 16 ICU nurses | Describe the ICU nurses’ experiences of working in teams during the Covid-19 pandemic | 4 ICUs in Sweden | 3 themes: losing the security of the ICU team; having time to adapt and finding structure for collaborative work; gaining professional growth through adapting collaborative work to contextual challenges |
| Fernández-Castillo  (2021) | Spain | Qualitative research; semi-structured videocall interviews; template analysis model of Brooks | 17 ICU nurses | Explore and describe the experiences and perceptions of nurses working in an ICU during the COVID-19 global pandemic | ICU at a tertiary teaching hospital in southern Spain | 4 themes: providing nursing care; psychosocial aspects and emotional lability; resources management and safety; professional relationships and fellowship |
| Fredholm  (2024) | Sweden | Qualitative research; semi-structured videocall interviews; template analysis model of Brooks; deductive content analysis | 15 CCNs, all with specialized training certificates in critical care and master's degrees in nursing | Analyze CCN ex- periences of supervision of nurses without training in ICU during the COVID-19 pandemic, and to analyze these experiences with the help of the salutogenic concept sense of coherence. | ICUs in multiple hospitals in Sweden, covering university hospitals, county hospitals, and local hospitals | 3 themes: comprehensibility; manageability; meaningfulness |
| Geltmeyer  (2022) | Belgium | Rapid qualitative descriptive research; semi-structured interview; thematic Analysis | 17 ICU nurses; 12 supporting nurses (mixed sample) | Investigate the perceptions and experiences of ICU teams regarding the implementation of a new mixed nursing care model during the COVID-19 pandemic | ICU of a tertiary university affiliated hospital in Flanders, Belgium | 1 important common theme: ensuring safe, high-quality care |
| Gordon  (2021) | America | Descriptive qualitative study; semi-structured interview | 11 ICU nurses | Explore the experiences of ICU nurses in the United States in caring for patients with COVID-19 | ICUof a community hospital (St. David's Round Rock Medical Center) in central Texas, USA. | 5 main themes (subthemes): **emotions experienced** (anxiety/stress, fear, helplessness, worry, and empathy); **physical symptoms** (sleep disturbances, headaches, discomfort, exhaustion, and breathlessness); **care environment challenges** (surrogate, inability to provide human comforting connection, patients dying, personal protective equipment (PPE), isolation, care delay, changing practice guidelines, and language barrier); **social effects**: (stigma, divergent healthcare hero perception, additional responsibilities, strained interactions with others, and isolation/loneliness); **short term coping strategies** ;( coworker support, family support, distractions, mind/body wellness, and spiritualty/faith) |
| Griffin  (2024) | the United Kingdom | Semi-structured interview | 54 critical care nurses | What are the experiences and implications of moral injury in critical care nursing during the pandemic? | UK (mainly in the intensive care units of NHS hospitals, with some in Ireland) | 3 themes: unsafe staffing levels; inadequate equipment; inability to provide patients with a dig- nified death |
| Holtz  (2025) | America | Qualitative descriptive approach; secondary data analysis; templating thematic analysis | 29 nurses who worked in intensive care units and emergency departments. | Explore the perceptions of organizational betrayal by the healthcare system among direct care nurses in ICU and emergency departments during the COVID-19 pandemic and to identify its underlying causes | Two acute care academic hospitals designated as "Magnet" hospitals in Pennsylvania and Missouri, USA. | 3 themes: inconsistent leadership in response to uncertainty and constraints; financial and human resource challenges; persistent residue of perceived ongoing organizational betrayal |
| Kagan  (2022) | Israel | Focus groups | 15 senior managerial nurses; 145 ICU nurses (mixed sample) | Understand staff perspectives on adaptive models of care employed in ICUs during the pandemic | 5 ICUs in a large tertiary medical center in Israel | 2 themes: challenges of the COVID-19 pandemic; positive aspects of the COVID-19 pandemic |
| Karataş Baran  (2024) | Turkey | Empirical phenomenological approach; semi-structured interview; 7‐step method of Colaizzi | 50 ICU nurses | Describe the working experiences, feelings, and thoughts of nurses working in the ICU during the COVID-19 pandemic | A hospital in Ankara, Türkiye | 6 themes: backbone of the health system nursing; professional achievements; difficulties encountered; support needs and expectations; changes in emotions; private life. |
| Khorasani  (2023) | Iran | Conventional content analysis | 12 ICU nurses | Explain the nurses’ experience of the organizational climate of the COVID-19 ICUs | 3 teaching hospitals in Isfahan, Iran | 3 themes: positive climate of attachment and professional commitment; emotional resonance in the work environment; supportive environment of the organization |
| Kilcommons  (2025) | Canada | Descriptive interpretive qualitative design; thematic analysis | 19 registered ICU nurses | Determine the factors most likely to promote nurse retention in the context of the COVID-19 pandemic. | Internal medicine/surgical ICU at a Level 3 medical center in Alberta, Canada | 4 themes: organizational resources and scheduling; interpersonal factors; mental health support; training and career advancement |
| Littlemouse  (2024) | America | Descriptive qualitative study; watson's theory of caring science; phenomenological approach | 11 ICU nurses | ICU nurses’ lived experiences caring for patients with COVID-19 while trying to engage in self-care and care for their families | ICU in a large hospital in the western United States | 4 themes: love for ICU nursing; not feeling supported by administration; ICU nurse health; ICU nurses feeling disrespected |
| Mailani  (2025) | Indonesia | Hermeneutic phenomenological approach | 20 ICU nurses | Explore the experience of indonesian nurses who work in ICU for COVID‐19 patients | ICUs of eight COVID-19 referral tertiary hospitals in seven major cities in Indonesia | 4 themes: the pleasing and bad feelings; new challenges of working; nursing professional growth; nurse resource management for COVID‐19 |
| McGillis Hall  (2023) | Canada | Descriptive interpretive qualitative design; focus group interviews; thematic analysis | 45 registered ICU nurses | Understand staff perspectives on adaptive models of care employed in ICUs during the pandemic | ICUs in an academic health network composed of 8 hospitals in the Toronto area of Canada. | 3 themes: effective elements; challenges experienced; areas of consideration for future implementation moving forward |
| Molala  (2024) | South Africa | Qualitative, exploratory, descriptive, and contextual research design; phenomenological approach | 15 ICU nurses | Explores the lived experiences of professional nurses caring for COVID-19 ICU patients in private hospitals in Gauteng, South Africa | The 5 largest private hospitals under the same private hospital group in Gauteng Province, South Africa | 3 themes: abrupt transition from normality to the COVID-19 pandemic; experienced isolation from family, community, and nursing management; feelings of satisfaction and gratitude for teamwork and learning |
| Montgomery  (2021) | the United Kingdom | Qualitative research; semistructured telephone interviews; rapid analysis | 8 ICU nurses; 13 redeployed nurses (mixed sample) | Understand NHS staff experiences of working in critical care during the first wave of the COVID-19 pandemic in the UK | 4 hospitals in the UK | 7 themes: fear and dread of COVID-19; purpose and duty; ordeals in critical care; isolation and the ‘COVID-19 bubble’; learning and creativity; teamwork; donning and doffing |
| Moradi  (2021) | Iran | Qualitative descriptive study; semi-structured face-to-face interviews; content analysis method | 17 ICU nurses | Explore the challenges experienced by ICU nurses throughout the provision of care for COVID-19 patients | ICU of a COVID-19 Center in Urmia, Iran | 4 themes: organization's inefficiency in supporting nurses; physical exhaustion; living with uncertainty; psychological burden of the disease |
| Parissopoulos  (2023) | Greece | Phenomenological approach; semi-structured in-depth interviews | 11 ICU nurses; 8 nursing managers (mixed sample) | Explore the experience of caring through the narratives of ICU nurses in Greece | 18 bed comprehensive ICU in a public teaching hospital in Athens, Greece | 4 themes: being "proximal"; "co-present"and caring with empathy; being “responsible” for your patient and negotiating with the doctors; technology and "fighting with all you’ve got"; "not being kept informed" and disappointment |
| Pogoy  (2021) | United Arab Emirates | Qualitative Husserlian phenomenological approach | 8 OFW ICU nurses | Explore the meaning of OFW nurses’ lived experiences caring for critically ill patients with COVID-19 | Multiple hospitals in Dubai, UAE | 4 themes: challenges during the pandemic; patient care during COVID-19; adapting to change; resilience amidst the pandemic |
| Pountney  (2023) | the United Kingdom | Qualitative design; semi-structured online interview; thematic analysis; | 10 nurses working in the pediatric intensive care unit | Understand PCC nurses' lived experience of working during COVID-19 to determine the impact it had on their well-being | 6 pediatric ICUs in the UK | 5 themes: challenges of working in PPE; adapting to redeployment to adult intensive care; changes to staff working relationships; being unable to attain work-life balance; unprocessed traumatic experiences of working in COVID-19 |
| Putruttanamaneekun  (2025) | Thailand | Qualitative, phenomenological study; Gadamer's philosophical hermeneutics | 15 ICU nurses | Explore the lived experiences of ICU nurses caring for critically ill patients infected with COVID-19 in Thailand | A university hospital in central Thailand | 3 themes: Being a hero in the COVID-19 war; feeling frustrated in life and having no place to go; acknowledging growing up in the nursing career |
| Rad  (2023) | Iran | Qualitative study; grounded theory approach | 14 ICU nurses; 4 head nurse; 1matron; 1 physician;1 psychologist (mixed sample) | Explore ICU nurses’ experiences of caring for patients with COVID-19 | ICUs at selected hospitals in Sabzewar, Neshapur, and Mashhad, Iran | 4 themes: ward overcrowding; changing patterns; complexity of care; feeling exhausted |
| Rosa  (2022) | Italy | Phenomenological study; Semi-structured interview | 16 ICU nurses | Investigate the experience lived by nurses who worked in an ICU during the COVID-19 pandemic | ICU of Northern Italian Hospital | 5 themes: pride, isolation, and fear; teamwork and organisation; moral/ethical aspect; true heroes; dignity. |
| Saidkhani  (2024) | Iran | Conventional content analysis; semistructured interview; | 20 ICU nurses | Explain the experience of ICU nurses during the COVID‑19 crisis | The ICU of the university hospital affiliated with Jundishapur Medical University in Ahvaz, Iran | 1 theme: growth under pressur;  8 main categories: psychological crisis; physical exhaustion; family conflicts; complex care; professional development; expertise; life enrichment; full support |
| Scott  (2023) | the United Kingdom | Naturalistic inquiry; semi-structured individual interviews; reflexive thematic analysis | 10 registered ICU nurses;2 student nurses; 3 doctors; 2 allied health professionals (mixed sample) | Examine whether the sources of moral distress are similar, or different, to those that commonly occur in critical care departments | ICU of an Emergency Hospital in Northern England | 5 themes: counterintuitive care; tough days; not the usual standard; personal and family safety; consequence of moral distress |
| Sezgin  (2022) | Turkey | Descriptive qualitative study; thematic analysis; semi-structured interview | 5 ordinary ICU nurses | Describe the experiences of ICU nurses who provided care to COVID-19 patients and their perceptions towards the disease and their work conditions during the pandemic | 7 hospitals in Istanbul, Türkiye (5 public hospitals and 2 private hospitals) | 5 themes: death and fear of death; impact on family and social lives; nursing care of COVID-19 patients; changing perceptions of their own profession: empowerment and dissatisfaction; experiences and perceptions of personal protective equipment and other control measures |
| Sibiya  (2025) | South Africa | Exploratory, qualitative descriptive study; thematic analysis; Semi-structured interview | 21 ICU nurses | Explore the experiences, specifically the ethical dilemmas and moral distress, of CCNs working in South African hospitals | Gauteng Province, South Africa | 5 themes: deep caring for patients versus pragmatic compromises; ambivalence  about the Nursing Oath; emotional labour (moral distress), uncertainty and fear; lack of  investment and appreciation; navigating resource constraints |
| Slettmyr  (2023) | Sweden | Descriptive phenomenological study; malterud's systematic text condensation; semi-structured interview | 14 from adult ICU and 6 from pediatric ICU | Understand the lived experience of altruism and sacrifices among Swedish nurses working in ICU during the COVID-19 pandemic | 2 city hospitals in Stockholm, Sweden | 4 themes: the work situation changed from 1 day to another; adapting to the chaotic situation; being confronted with ethical and moral challenges; the importance of supporting each other |
| Slettmyr  (2025) | Sweden | Phenomenological hermeneutical method; interviewed individually via telephone, online or in-person | 11 nurses who previously worked in the ICU but have since resigned | Explore ICU nurses’ course towards making the decision to resign from work in the ICU following the COVID-19 pandemic | Multiple hospitals across Sweden | 3 themes: to give it all and yet feel insufficient; to experience togetherness and yet feel lonely; to prioritise others and yet need to eventually prioritise oneself |
| Stayt  (2022) | the United Kingdom | Qualitative interview study; Semi-structured interview; thematic analysis | 11 ICU nurses; 8 redeployed nurses (mixed sample) | Explore registered nurses' experiences of patient safety in ICU during COVID-19 | - | 2 themes: on a war footing; doing the best we can |
| Wu  (2023) | China | Phenomenological qualitative design; semi-structured individual telephone interviews; Colaizzi's 7-step method | 12 ICU nurses | Explore the psychological experience and coping methods of nurses exposed to workplace violence | A designated hospital in Wuhan | 3 themes: full of negative emotions; facing challenges and danger; coping methods |
| Yin  (2022) | China | Qualitative study; semi-structured interview; thematic analysis | 6 ICU nurses; 8 doctors  (mixed sample) | Describe the different phases of psychological distress of FHWs during the early stage of the COVID-19 pandemic | Zhengzhou University First Affiliated Hospital, Zhengzhou City, Henan Province, China | 5 themes: the mobilisation period: a sense of responsibility with worries; the preparation period: worries, fears and doubts about the epidemic; the transitional period: complex and diverse psychological feelings; the adaptation period: self-adjustment and help from external support; the reflection period: a reflection on life and nature |
| Yousefi  (2023) | Iran | Qualitative study; conventional content analysis | 17 ICU nurses | Investigate the occupational challenges experienced by ICU nurses in caring for patients with COVID-19 | COVID-19 specific ICU in a large hospital in eastern Iran | 6 themes: payment system; human resource management; consumable resource supply; psychological and ethical distress; personal or family problems; staff motivation and welfare issues |

CCNs, critical care nurses; COVID-19, CoronaVirus Disease 2019; ICU, intensive care unit; OFW, Overseas Filipino Workers; PPE, Personal Protective Equipment; SARS-CoV-2, Severe Acute Respiratory Syndrome Coronavirus 2; UAE, United Arab Emirates.

**Table S5 Joanna Briggs Institute critical appraisal of included studies.**

| First Author, year | | Q1 | Q2 | Q3 | Q4 | Q5 | Q6 | Q7 | Q8 | Q9 | Q10 | Result (%) |
| --- | --- | --- | --- | --- | --- | --- | --- | --- | --- | --- | --- | --- |
| Aamodt (2025) | | Y | Y | Y | Y | Y | Y | Y | Y | Y | Y | 20/20(100%) |
| Al Haddad (2024) | | Y | Y | Y | Y | Y | Y | Y | Y | Y | Y | 20/20(100%) |
| Alzailai (2023) | | Y | Y | Y | Y | Y | Y | Y | Y | N | Y | 18/20(90%) |
| Askar (2023) | | Y | Y | Y | Y | Y | Y | Y | Y | Y | Y | 20/20(100%) |
| Aydin (2022) | | Y | Y | Y | Y | Y | Y | Y | Y | Y | Y | 20/20(100%) |
| Baran (2024) | | Y | Y | Y | Y | Y | N | U | Y | Y | Y | 17/20(85%) |
| Besen (2023) | | Y | Y | Y | Y | Y | N | U | Y | Y | Y | 17/20(85%) |
| Cadge (2021) | | Y | Y | Y | Y | Y | N | U | Y | Y | Y | 17/20(85%) |
| Chegini (2021) | | Y | Y | Y | Y | Y | N | U | Y | Y | Y | 17/20(85%) |
| Christianson (2022) | | Y | Y | Y | Y | Y | N | U | Y | Y | Y | 17/20(85%) |
| Costa (2023) | | Y | Y | Y | Y | Y | N | U | Y | N | Y | 17/20(85%) |
| Costa (2023) | | Y | Y | Y | Y | Y | N | U | Y | Y | Y | 17/20(85%) |
| Credland (2024) | | Y | Y | Y | Y | Y | Y | Y | Y | Y | Y | 20/20(100%) |
| Díaz-Agea (2022) | | Y | Y | Y | Y | Y | N | U | Y | Y | Y | 17/20(85%) |
| Digby (2023) | | Y | Y | Y | Y | Y | N | U | Y | Y | Y | 17/20(85%) |
| Eckerblad (2025) | | Y | Y | Y | Y | Y | N | U | Y | Y | Y | 17/20(85%) |
| Fernández-Castillo (2021) | | Y | Y | Y | Y | Y | N | U | Y | Y | Y | 17/20(85%) |
| Fredholm (2024) | | Y | Y | Y | Y | Y | N | U | Y | Y | Y | 17/20(85%) |
| Geltmeyer (2022) | | Y | Y | Y | Y | Y | Y | Y | Y | Y | Y | 20/20(100%) |
| Gordon (2021) | | Y | Y | Y | Y | Y | N | U | Y | N | Y | 15/20(75%) |
| Griffin (2024) | | Y | Y | Y | Y | Y | N | U | Y | Y | Y | 17/20(85%) |
| Holtz (2025) | | Y | Y | Y | Y | Y | N | U | Y | N | Y | 15/20(75%) |
| Kagan (2022) | | Y | Y | Y | Y | Y | N | U | Y | Y | Y | 17/20(85%) |
| Karataş Baran (2024) | | Y | Y | Y | Y | Y | N | U | Y | Y | Y | 17/20(85%) |
| Khorasani (2023) | | Y | Y | Y | Y | Y | N | U | Y | Y | Y | 17/20(85%) |
| Kilcommons (2025) | | Y | Y | Y | Y | Y | Y | Y | Y | Y | Y | 20/20(100%) |
| Littlemouse (2024) | | Y | Y | Y | Y | Y | Y | Y | Y | Y | Y | 20/20(100%) |
| Mailani (2025) | | Y | Y | Y | Y | Y | N | U | Y | Y | Y | 17/20(85%) |
| McGillis Hall (2023) | | Y | Y | Y | Y | Y | N | U | Y | Y | Y | 17/20(85%) |
| Molala (2024) | | Y | Y | Y | Y | Y | N | U | Y | Y | Y | 17/20(85%) |
| Montgomery (2021) | | Y | Y | Y | Y | Y | N | U | Y | Y | Y | 17/20(85%) |
| Moradi (2021) | | Y | Y | Y | Y | Y | N | U | Y | N | Y | 15/20(75%) |
| Parissopoulos (2023) | | Y | Y | Y | Y | Y | N | U | Y | Y | Y | 17/20(85%) |
| Pogoy (2021) | | Y | Y | Y | Y | Y | N | U | Y | Y | Y | 17/20(85%) |
| Pountney (2023) | | Y | Y | Y | Y | Y | N | U | Y | Y | Y | 17/20(85%) |
| Putruttanamaneekun (2025) | | Y | Y | Y | Y | Y | N | U | Y | Y | Y | 17/20(85%) |
| Rad (2023) | | Y | Y | Y | Y | Y | Y | Y | Y | Y | Y | 20/20(100%) |
| Rosa (2022) | | Y | Y | Y | Y | Y | N | U | Y | Y | Y | 17/20(85%) |
| Saidkhani (2024) | | Y | Y | Y | Y | Y | N | U | Y | Y | Y | 17/20(85%) |
| Scott (2023) | | Y | Y | Y | Y | Y | N | U | Y | Y | Y | 17/20(85%) |
| Sezgin (2022) | | Y | Y | Y | Y | Y | Y | Y | Y | Y | Y | 20/20(100%) |
| Sibiya (2025) | | Y | Y | Y | Y | Y | Y | Y | Y | Y | Y | 20/20(100%) |
| Slettmyr (2023) | | Y | Y | Y | Y | Y | Y | Y | Y | Y | Y | 20/20(100%) |
| Slettmyr (2025) | | Y | Y | Y | Y | Y | N | U | Y | Y | Y | 17/20(85%) |
| Stayt (2022) | | Y | Y | Y | Y | Y | N | U | Y | Y | Y | 17/20(85%) |
| Wu (2023) | | Y | Y | Y | Y | Y | N | U | Y | Y | Y | 17/20(85%) |
| Yin (2022) | | Y | Y | Y | Y | Y | N | U | Y | Y | Y | 17/20(85%) |
| Yousefi (2023) | | Y | Y | Y | Y | Y | N | U | Y | Y | Y | 17/20(85%) |
|  | Domains:  1. Congruity between the stated philosophical perspective and the research methodology 2. Congruity between the research methodology and the research question or objectives 3. Congruity between the research methodology and the methods used to collect data 4. Congruity between the research methodology and the representation and analysis of data  5. There is congruence between the research methodology and the interpretation of results  6. Locating the researcher culturally or theoretically  7. Influence of the researcher on the research, and vice-versa, is addressed  8. Representation of participants and their voices 9. Ethical approval by an appropriate body 10. Relationship of conclusions to analysis or interpretation of the data  Code: Y: Yes N: No U: Unclear | | | | | | | | | | | |


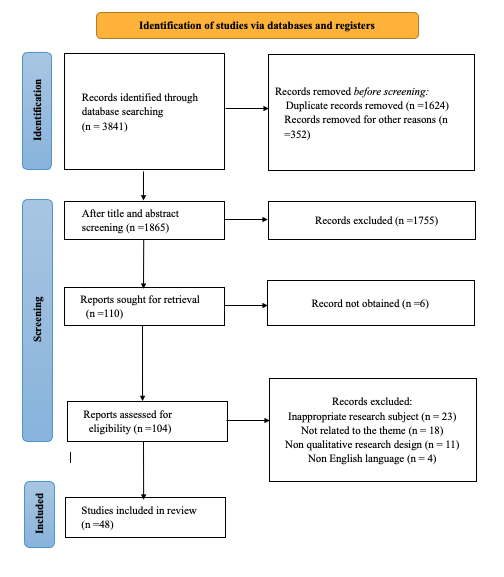

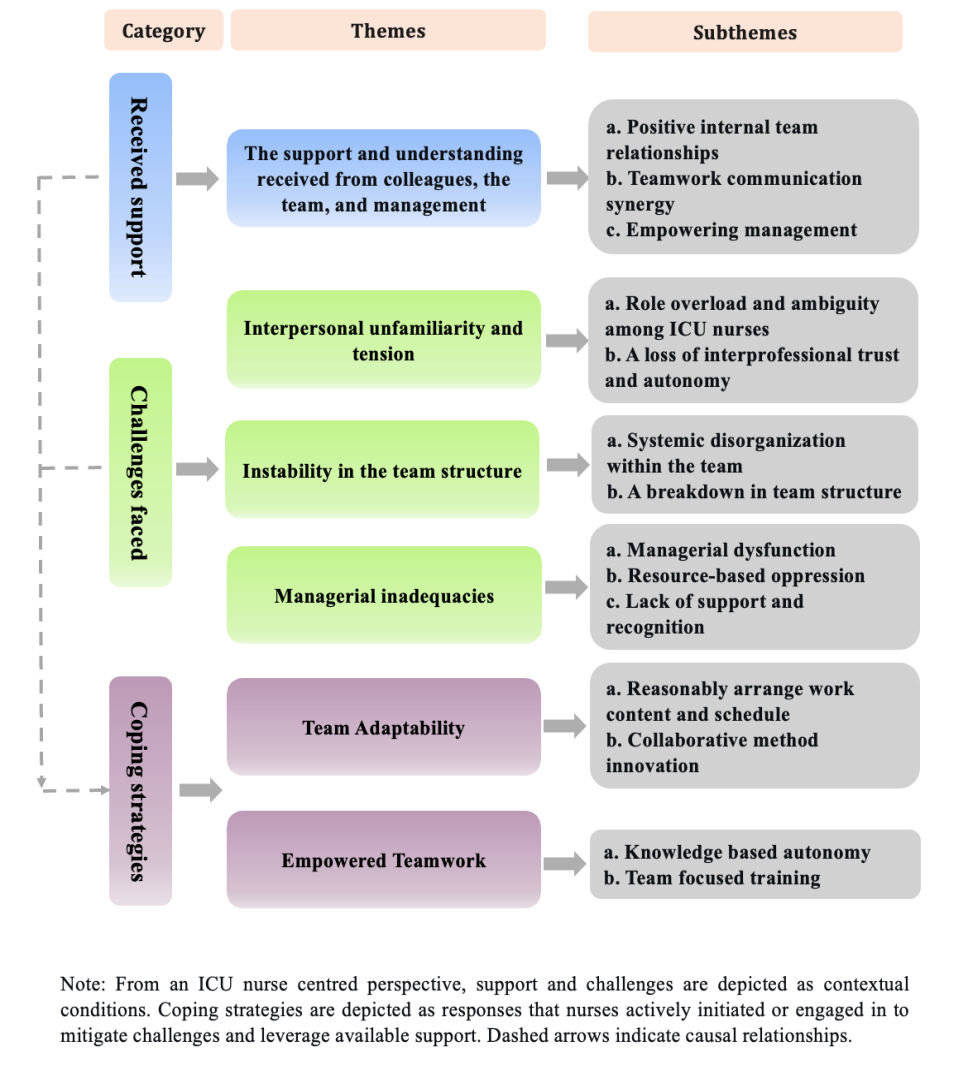


**Figure S1** Literature screening process **Figure S2** Overview map of themes and subthemes
